# Unmasking host and microbial strategies in the *Agrobacterium*-plant defense tango

**DOI:** 10.3389/fpls.2015.00200

**Published:** 2015-03-31

**Authors:** Elizabeth E. Hwang, Melinda B. Wang, Janis E. Bravo, Lois M. Banta

**Affiliations:** Thompson Biology Lab, Department of Biology, Williams CollegeWilliamstown, MA, USA

**Keywords:** immunity, pathogen, *Agrobacterium*, hormones, actin, trafficking, temperature, light

## Abstract

Coevolutionary forces drive adaptation of both plant-associated microbes and their hosts. Eloquently captured in the Red Queen Hypothesis, the complexity of each plant–pathogen relationship reflects escalating adversarial strategies, but also external biotic and abiotic pressures on both partners. Innate immune responses are triggered by highly conserved pathogen-associated molecular patterns, or PAMPs, that are harbingers of microbial presence. Upon cell surface receptor-mediated recognition of these pathogen-derived molecules, host plants mount a variety of physiological responses to limit pathogen survival and/or invasion. Successful pathogens often rely on secretion systems to translocate host-modulating effectors that subvert plant defenses, thereby increasing virulence. Host plants, in turn, have evolved to recognize these effectors, activating what has typically been characterized as a pathogen-specific form of immunity. Recent data support the notion that PAMP-triggered and effector-triggered defenses are complementary facets of a convergent, albeit differentially regulated, set of immune responses. This review highlights the key players in the plant’s recognition and signal transduction pathways, with a focus on the aspects that may limit *Agrobacterium tumefaciens* infection and the ways it might overcome those defenses. Recent advances in the field include a growing appreciation for the contributions of cytoskeletal dynamics and membrane trafficking to the regulation of these exquisitely tuned defenses. Pathogen counter-defenses frequently manipulate the interwoven hormonal pathways that mediate host responses. Emerging systems-level analyses include host physiological factors such as circadian cycling. The existing literature indicates that varying or even conflicting results from different labs may well be attributable to environmental factors including time of day of infection, temperature, and/or developmental stage of the host plant.

## Overview

At its most basic, any form of immunity must distinguish between self, or beneficial, and harmful non-self interactions. In animals, adaptive immunity is delegated to specialized immune cells that undergo selection to recognize new pathogens and mount specific, targeted defenses more rapidly during a second attack. This adaptive immunity is only possible because innate immunity, consisting of evolutionarily ancient, non-specific and rapidly mobilized defenses, staves off – or eliminates – the pathogen while adaptive immunity is activated. Innate immune responses are triggered by highly conserved “pathogen-associated molecular patterns,” or PAMPs, that are widely shared within distinct classes of pathogens, such as bacteria or fungi. Due to their structural and sequence-level evolutionary conservation, PAMPs are often found in non-pathogenic microbes as well and hence are also called microbial-associated molecular patterns, or MAMPs. These PAMPs/MAMPs elicit a set of defense mechanisms tailored to the type of microbe perceived (Ausubel, 2005).

Plants lack specialized immune cells, but have a robust and sophisticated system of innate defenses. Extra- and intra-cellular receptors detect microbial presence and trigger signal transduction pathways that lead to immediate physiological changes. These include the production of damaging reactive oxygen species (ROS), extracellular alkalization, Ca^2+^ fluxes, callose deposition, seedling growth inhibition, stomate closure, and localized programmed cell death [hypersensitive response (HR); Boller and Felix, 2009]. One of the earliest signal transduction events regulating these responses is the mitogen-activated protein kinase (MAPK) pathway, which modulates defense gene expression (Asai et al., 2002). Hormone biosynthesis genes, notably for salicylic acid (SA), jasmonate (JA) and ethylene, are among those expressed, and play a crucial role in plant defenses. These hormones can induce systemic defenses throughout the plant to slow or prevent the pathogen from spreading beyond the infection site. They also upregulate hormone-specific defense genes further downstream and in their volatile forms, can even signal to neighboring plants (Shulaev et al., 1997).

Two arms of the plant innate immune system control the selective activation of these responses: effector-triggered immunity (ETI) and PAMP (or pattern)-triggered immunity (PTI; also called MTI, or MAMP-triggered immunity). Unlike PAMPs or MAMPs, effectors are secreted only by specific bacterial strains and are highly polymorphic (Spoel and Dong, 2012). Effectors are generally virulence-promoting factors that typically suppress host defenses. Particular plant genomes encode R proteins that specifically recognize and bind bacterial effectors to reduce their efficacy, decreasing plant susceptibility (Jones and Dangl, 2006).

A unifying theme of this review is the interplay between *Agrobacterium tumefaciens* and the defense responses mounted by its hosts. *Agrobacterium*, the causative agent of crown gall disease, is best known for its ability to genetically transform host plants by delivering a portion (the “T-DNA”) of its tumor-inducing (Ti) plasmid or a foreign DNA construct that has been inserted into a Ti plasmid-derived vector (Lacroix and Citovsky, 2013a). A number of studies (e.g., Ditt et al., 2005; Zipfel et al., 2006; Tie et al., 2012; Zhang et al., 2013b) in a range of host species including rice, ryegrass, and *Arabidopsis* *thaliana* indicate that *Agrobacterium*-elicited defenses limit transformation efficiency. However, unlike several other well-characterized phytopathogens*, Agrobacterium* does not appear to incite an HR on most host species. Consistent with this observation, there is growing evidence that *Agrobacterium* modulates host defenses, at least in part by regulating hormone accumulation, although the mechanism(s) mediating this host manipulation are not yet known. Given the relative paucity of published studies on the defenses affected by *Agrobacterium*, our goal here is to synthesize for the reader those facets of the host response that appear most relevant to *Agrobacterium* infection. Because there are no known *Agrobacterium*-encoded effectors or cognate host R proteins, we focus primarily on PAMP-triggered immune signaling and its downstream consequences. Nonetheless, in light of recent arguments questioning the distinction between PTI and ETI (see PTI and ETI: A False Distinction?), we briefly consider ETI as well before reviewing in detail the hormonal regulatory pathways (particularly SA) that appear to be a critical target for agrobacterial counter-defense strategies. Finally, we highlight both host physiological and environmental factors that may impact the outcome of the host-*Agrobacterium* interaction.

## Pathogen Elicitors and Host Recognition/Response Systems

### PAMP Perception and PTI

PAMP-triggered immunity is elicited by highly conserved molecular features, such as bacterial flagellin. These patterns are specific epitopes derived from molecular structures that are essential for microbial fitness. In general, evolutionary selective pressure prevents the loss or modification of the PAMPs and, in theory, distinguishes PAMPs from host-specific pathogen-derived effectors. As universal harbingers of microbial presence, known PAMPs predictably include a number of cell wall components such as peptidoglycan, lipopolysaccharides, and fungal chitin (Felix et al., 1999; Gust et al., 2007; Miya et al., 2007; Erbs et al., 2008; Thomma et al., 2011). The two best-characterized bacterial PAMPs are peptides derived from flagellin (flg22) and the elongation factor EF-Tu (elf18; Felix et al., 1999; Kunze et al., 2004). Flagellin and more generally, pathogen motility, play key roles in pathogenesis, as chemotaxis and entry into the host are often essential early in infection (Josenhans and Suerbaum, 2002). EF-Tu is the most abundant protein found in many bacteria experiencing rapid growth (Furano, 1975) and is released into the extracellular space upon disruption of bacterial cell membrane integrity (Zipfel et al., 2006; Nicaise et al., 2009).

PAMPs and MAMPs are perceived by the extracellular domains of plant pattern recognition receptors (PRRs), typically receptor-like kinases that trigger downstream kinase-dependent signaling pathways. No intracellular PRRs have yet been found (Thomma et al., 2011). In *Arabidopsis*, the flagellin receptor FLS2 and the EF-Tu receptor (EFR) detect subnanomolar concentrations of flg22 and elf18, respectively (Chinchilla et al., 2006; Zipfel et al., 2006). The presence of EFR only in *Brassicaceae* (Boller and He, 2009) suggests that it is evolutionarily younger than FLS2 (Nekrasov et al., 2009; Saijo et al., 2009). The demonstrated success in conferring resistance to *Agrobacterium* and other pathogens by introducing the *EFR* gene from *Arabidopsis* into *Nicotiana benthamiana* and tomato (*Solanum lycopersicum*) raises the possibility of engineering broad-spectrum bacterial resistance by heterologous expression of PRRs in vulnerable crops (Lacombe et al., 2010).

Significantly, some pathogens exhibit enough divergence in their PAMP sequences to avoid host detection (Boller and Felix, 2009). In particular, the *Arabidopsis* FLS2 is unable to perceive flagellin from *Agrobacterium* (Bauer et al., 2001). Instead, EFR appears to be the key determinant of susceptibility to *Agrobacterium* in *Brassicaeceae*. Co-inoculation with the elicitor peptide elf18 reduces transformation efficiency of the bacterium, while an *efr* mutant plant line exhibits enhanced susceptibility to infection and transgene transformation (Zipfel et al., 2006). *Agrobacterium* cell-wall derived peptidoglycans do elicit defense responses, including rapid increases in ROS and extracellular pH in *Arabidopsis*, albeit at much reduced levels as compared to those from the necrotrophic pathogen *Xanthomonas campestris* pathovar *campestris*; this difference may reflect the agrobacterial requirement to maintain host viability for successful transformation (Erbs et al., 2008).

### Effectors and ETI

Since microbes unwittingly elicit PTI with passive expression of conserved microbial patterns, successful infection often requires the secretion of virulence effectors to subvert those defenses (Jones and Takemoto, 2004). While many effectors act enzymatically, others decrease plant defenses by increasing transcription of genes that further down-regulate defense activation genes (Chisholm et al., 2006). Still other effectors, including the *Pseudomonas syringae* pv. *tomato* DC3000 Type III secretion system (T3SS)-delivered avirulence factors AvrPto and AvrPtoB, directly target PRRs for inactivation and suppress PTI signaling events, thus increasing host susceptibility to the incoming pathogen (Abramovitch et al., 2006; He et al., 2007; Zipfel, 2009).

Predictably, many plant hosts have evolved a second branch of immunity called ETI to detect these virulence-promoting effectors via polymorphic nucleotide-binding leucine-rich repeat (NB-LRR) or extracellular leucine-rich repeat (eLRR) proteins encoded by *R* genes (Jones and Dangl, 2006). ETI is also known as “gene-for-gene immunity” because R proteins have evolved to specifically detect and recognize particular pathogenic effectors (Chisholm et al., 2006). Some R proteins can also indirectly recognize the changes in host proteins targeted by pathogen effectors, a phenomenon initially articulated as the “guard hypothesis” (Jones and Takemoto, 2004). *R* gene-mediated resistance results in severe host defense activation, including a HR, or apoptosis at the infection site, in an effort to limit the pathogen’s spread throughout the plant and hence the development of disease (He et al., 2007).

Unlike MAMPs, effectors are not required for microbe survival, and are thus under strong evolutionary pressure to mutate and evade host plant detection by R proteins. This force similarly drives *R* genes to mutate to more successfully detect effectors. The “four phased ‘zigzag’ model” (Jones and Dangl, 2006) maps the dance between pathogen attack and plant defense. In this model, the plant’s detection of PAMPs triggers PTI, which is dampened by the pathogen’s secretion of effectors. These effectors (sometimes referred to as Avr proteins) are then recognized by plant R proteins, increasing plant defense via ETI, until mutated effectors can evade plant detection and successfully hamper plant defense activation (Jones and Dangl, 2006). The co-evolution or Red Queen-like relationship between effectors and R proteins drives the ‘zigzag’ as the plant or the pathogen temporarily gains the upper hand.

As noted above, there are no reports to date of a classic gene-for-gene mechanism of resistance in any host species to *Agrobacterium*. There is, however, mounting evidence that the pathogen has the capability to disable or dampen defenses. Ditt et al. (2006) reported no difference in gene expression between *Agrobacterium*-infected and mock-infected *Arabidopsis* at 4–24 h post-infection (hpi), although their microarray analysis did reveal distinct sets of up-regulated defense genes, as well as down-regulated cell-proliferation genes, at 48 hpi. In contrast, other studies (e.g., Veena et al., 2003; Lee et al., 2009; Zhang et al., 2015) uncovered a variety of alterations in the host transcriptome at earlier time points. Given the rapidity of the basal defenses described here, it seems likely that Ditt et al. (2006) missed many of the changes in gene expression that may well have returned to pre-infection levels by 4 hpi. Using subtractive hybridization and macroarray analysis for expression profiling, Veena et al. (2003) noted that defense gene induction in tobacco BY2 suspension cells was suppressed at 30–36 hpi by a strongly virulent *Agrobacterium* strain but not by a Ti plasmid-deficient strain, although early defense gene induction (3–6 hpi) appeared to be largely similar between the two. Consistent with the observed capacity of the pathogen to suppress accumulation of ROS within 3 h of infection (Lee et al., 2009), a catalase-deficient mutant is highly attenuated for virulence (Xu and Pan, 2000). Somewhat paradoxically, Anand and Mysore (2013) found that RAR1, a plant protein required for R-gene mediated resistance to fungal pathogens, contributes to efficient *Agrobacterium* transformation. Finally, hormones produced by *Agrobacterium* block the HR that would normally result from subsequent infection with *P. syringae* pv. *phaseolicola* (Robinette and Matthysse, 1990) and enhance host susceptibility by suppressing expression of an infection-inhibiting transcription factor (Sardesai et al., 2013). Compared to the plethora of data on effectors produced by other pathogens, especially those with T3SS, we still know very little about the mechanisms *Agrobacterium* employs to thwart host defenses. The examples cited here hint at a diversified portfolio of strategies that may fail to conform to the canonical effector-R gene-mediated duel for dominance (Anderson et al., 2010). Instead, it seems reasonable to hypothesize that molecules delivered via the Ti-plasmid and/or chromosomally borne secretion systems in *Agrobacterium* could promote host transformation by repressing defense activation (or inducing expression of defense repressors). Similar to ETI, such *Agrobacterium*-derived saboteurs might in turn induce additional host responses.

### PTI and ETI: A False Distinction?

Several lines of evidence have recently called into question the notion of clear temporal, evolutionary and structural distinctions between PTI and ETI (Qi et al., 2011; Thomma et al., 2011). For example, the *Arabidopsis* proteins RPM1, RPS2, and RPS5 were identified as R proteins that indirectly detect effector activity on the plant protein RIN4 (Jones and Takemoto, 2004), a regulator of PAMP-triggered responses (Kim et al., 2005). Qi et al. (2011) recently demonstrated that RPM1, RPS2, and RPS5 also physically interact with FLS2 to trigger PTI. Moreover, certain PAMPs such as Ax21 and Pep-13 from *Xanthomonas oryzae* pv.* oryzae* and the *Phytophthora* species, respectively, are narrowly distributed and conserved in only a few strains of pathogens, characteristics classically attributed to effectors rather than PAMPs (Thomma et al., 2011).

Transcriptome analysis of inoculated plants also indicates that PAMP- and ETI may regulate similar sets of defense responses (Tsuda and Katagiri, 2010). Navarro et al. (2004) found overlap between *Arabidopsis* flg22-induced genes and their *P. syringae* effector-induced tobacco orthologs, though the extent of overlap was weak and diminished over time. Parallels in the early stages of the response indicate that both PAMPs and effectors may initially trigger a common signaling mechanism(s). Indeed, Boller and Felix (2009) have argued that there exists a single, convergent innate immune system whose kinetics and strength of response are fine-tuned depending on the type of perceived ligand (e.g., flg22). From this perspective, responses are ligand-dependent and an elicitor cannot be categorized as a PAMP or an effector based solely on its evolutionary conservation or role in virulence.

### Events Downstream of PRR-Mediated Perception of Bacterial Pathogens, and their Exploitation by *Agrobacterium*

#### Signal Transduction Cascades and Early Downstream Responses

Despite differences between FLS2 and EFR in mechanisms of biogenesis and desensitization to be discussed below, stimulation of *Arabidopsis* seedlings by flg22 or elf18 shows striking similarities in physiological responses and in the genes induced or repressed (Zipfel et al., 2006). For example, both peptides rapidly induce extracellular alkalization (Felix et al., 1999; Kunze et al., 2004), which would be predicted to enhance host resistance by attenuating the acid-dependent activation of the *Agrobacterium* virulence genes required for T-DNA transfer (Lacroix and Citovsky, 2013a). One mechanism that explains these overlapping responses is the use of a shared signaling pathway, which has been explored at two non-mutually exclusive levels: that of an adaptive co-receptor, and that of a shared MAPK signaling cascade.

The strong similarity in downstream responses led to a search for a common signaling element shared by EFR and FLS2. In particular, LRR receptor kinases belonging to the somatic-embryogenesis receptor-like kinase (SERK) family, particularly the Brassinosteroid receptor-associated kinase 1 (BAK1), have emerged as candidate co-receptors of EFR and FLS2. Using a reverse genetic screen to identify flg22-insensitive mutants, Chinchilla et al. (2007) discovered *bak1* mutants that were largely deficient in their flg22- and early elf18-induced ROS production but also showed a reduced response to brassinolide. Immunoprecipitation of MYC-tagged BAK1 *in vitro* in the presence of flg22 provided evidence for ligand-triggered formation of a FLS2-BAK1 complex. In a parallel experiment Heese et al. (2007) isolated the flg22-induced FLS2 complex and used MS/MS peptide sequencing to identify the co-immunoprecipitated BAK1 (also called SERK3). Roux et al. (2011) coimmunoprecipitated EFR with BAK1 and additionally showed that other SERK family members interact with EFR as well as with FLS2. Their results indicated that EFR can form strong complexes with several SERK family members (SERK1, SERK2, BAK1, and BKK1), while FLS2 complexes most strongly with BAK1, implying that functional redundancy between the SERK family members explains the observation that EFR is less dependent than FLS2 on BAK1 (Chinchilla et al., 2007; Roux et al., 2011; Schwessinger et al., 2011). Further experiments revealed that *bak1* null mutants are also impaired in their responses to lipopolysaccharides and peptidoglycans. Thus, BAK1 appears to act broadly as a co-receptor (Heese et al., 2007; Schulze et al., 2010). BAK1 also enhances FLS2 and EFR-mediated signaling by *trans*-phosphorylating the cytoplasmic kinase BIK1, a positive regulator of PTI shown to directly induce ROS production (Monaghan and Zipfel, 2012; Kadota et al., 2014). The recently identified BAK1-interacting receptor-like kinase BIR2 negatively regulates PTI by sequestering sub-pools of BAK1 from PRR interactions in the absence of a PAMP ligand, thereby controlling the availability of BAK1 to engage with its PRR partners (Halter et al., 2014a,b). The discovery of other critical co-receptors (e.g., BAK1-LIKE1, or BKK1), as well as other positive (e.g., SUPPRESSOR-OF-BIR1, or SOBIR1) and negative (e.g., BIR1) LRR-receptor-like kinase regulators has led to an appreciation of the cross-phosphorylation events that occur within an entire signaling complex, rather than an isolated BAK1-PRR interaction (Liebrand et al., 2014).

As its name implies, BAK1 was originally discovered to complex with a brassinosteroid receptor, BRI1 (Nam and Li, 2002). Brassinosteroids are plant hormones involved in cell growth and elongation, as well as in developmental processes including senescence (Clouse, 2011). *A priori*, it appeared plausible that a limiting pool of BAK1 could be responsible for the phenomenon of PAMP-triggered seedling growth inhibition, if FLS2/EFR-BAK1 complex formation competed with BR-mediated growth signals. However, elegant work by the Zipfel lab refuted this theory by showing brassinolide could inhibit PAMP-triggered responses without interfering with FLS2-BAK1 complex formation or downstream signaling (Albrecht et al., 2012). A forward genetic screen for elf18-insensitive mutants uncovered a new *bak1* mutant allele, *bak1-5*. Unlike the* bak1-4 bkk1-1* null mutant, the *bak1-5 bkk1-1* mutant shows far fewer pleiotropic effects and, in particular, normal brassinosteroid signaling and cell death control (Schwessinger et al., 2011). *bak1-5* is still severely impaired in characteristic PAMP-triggered responses, as well as in the ability to transphosphorylate BIK1 (Schwessinger et al., 2011).

Ligand binding to PRRs activates both MAPK and calcium-dependent protein kinase (CDPK) signaling cascades (Boudsocq et al., 2010; Tena et al., 2011). Asai et al. (2002) elucidated one such pathway, demonstrating that MEKK1, MKK4/5, and MPK3/6 mediate downstream responses upon flg22 perception. Chitin, peptidoglycan, and elf18 were also shown to induce MAPK activation. Interestingly, low concentrations of flg22 and elf18 act additively upon extracellular alkalization and MAPK activation, while high concentrations saturate both responses (Zipfel et al., 2006); these data indicate that a shared pool of MAP kinases may exist downstream of PAMP detection, although it remains unclear whether the MAPKs themselves are responsible for limiting the amplitude of defense responses. Upon reaching a duration and magnitude threshold, the MAPK cascade protein activation interfaces with calcium-activated pathways to activate further defense responses (Tena et al., 2011), including regulation of defense hormone synthesis, metabolite synthesis, stomatal closure, and antimicrobial compound synthesis (Meng and Zhang, 2013).

Another response mediated by the MAPK pathway upon PAMP activation is the induction of early defense genes such as *WRKY29* and *FRK1* (Asai et al., 2002). WRKY29 is part of the WRKY superfamily of transcription factors that affect pathogen defenses, wounding, senescence, and trichome development by interacting with W-box motifs (TTGAC) in the promoter regions of a large variety of target genes (Maleck et al., 2000). These regulatory proteins are characterized by a DNA-binding motif whose amino acid sequence (WRKYGQK) gives the family its name (Eulgem et al., 2000). The WRKY superfamily is very large, and WRKY proteins tend to interact and work in redundant and antagonistic roles depending on the type of pathogenic attack (Xu et al., 2006). While many WRKY factors promote resistance, many others suppress basal defenses to prevent deleterious effects on the host. In at least some cases, the same WRKY proteins that down-regulate PAMP-induced responses are inactivated upon R protein recognition of their cognate effectors during ETI, thus derepressing defense mechanisms (Kim et al., 2008). The complexity of the WRKY network is further embellished by multiple positive and negative feedback loops and feed-forward modules (Taj et al., 2014).

Given the complexity of the receptor network and the centrality of the MAPK pathway to PTI, it should come as no surprise that multiple pathogen-derived effectors target these various components. A complete catalog of such effectors is beyond the scope of this review (for a recent review, see Deslandes and Rivas, 2012). As one example, HopAI1 is delivered by the T3SS of *P. syringae* into the host cytoplasm, where it interacts physically with MPK3 and MPK6, deactivating the pathway via dephosphorylation (Zhang et al., 2007). Likewise, the *P. syringae* effector HopAO1 suppresses PAMP-induced resistance by reversing the ligand-induced tyrosine phosphorylation of EFR required for signal transduction (Underwood et al., 2007; Macho et al., 2014). The afore-mentioned *P. syringae* effector AvrPtoB physically associates with the FLS2/BAK1 complex, and acts as a ubiquitin ligase to target FLS2 for degradation (Gohre et al., 2008). In a classic tit-for-tat strategy, the host resistance protein Pto is able to inactivate the effector’s ligase domain, thus thwarting the pathogen’s attempt to block host immunity (Ntoukakis et al., 2009).

#### Exploitation of Early Host Defenses by *Agrobacterium*

Although there is no evidence that *Agrobacterium* can suppress the initial recognition by EFR and/or any associated co-receptors, both the downstream phosphorylation cascade and specific WRKY proteins are important targets for subversion by this pathogen. Within 5 min of exposure of *Arabidopsis* seedlings to *Agrobacterium*, the key defense modulators MPK3, MPK4, and MPK6 are phosphorylated (Djamei et al., 2007). One of the substrates of MPK3 is the stress-responsive transcription factor VIP1 (Pitzschke et al., 2009), which was initially identified as interacting with the *Agrobacterium* virulence protein VirE2 (Tzfira et al., 2001). Since MPK3-catalyzed phosphorylation of VIP1 results in its translocation to the nucleus, Djamei et al. (2007) proposed that nuclear localization of the interacting single-stranded DNA binding protein VirE2 would neatly serve to deliver the associated T-DNA as well. In this “Trojan horse” model, *Agrobacterium* co-opts the MAPK-mediated defense pathway it has triggered to ensure nuclear entry of its transgene “gift.” However, a very recent study from Shi et al. (2014) called this model into question by showing that under their experimental conditions neither the location of VirE2 nor host susceptibility to transformation correlate with, respectively, subcellular localization or abundance of VIP1. These authors’ alternative model posits that instead of shuttling the T-DNA to the nucleus, VirE2 promotes tumorigenesis by sequestering the low-abundance VIP1 in the cytoplasm, thus serving as a true effector to dampen the activity of this host defense-related transcription factor.

Expression of the* VIP1* gene is repressed in roots (but not in shoots) by WRKY17, a negative regulator of host defenses that may function to prevent over-reactive responses to pathogens. Mutant *Arabidopsis* deficient in *WRKY17* exhibit elevated levels of *Agrobacterium*-mediated transformation (Lacroix and Citovsky, 2013b), although they are more resistant than wild-type plants to *P. syringae* (Joumot-Catalino et al., 2006). This discrepancy between the responses of these two bacteria to a *wrky17* mutant illustrates a prevailing observation that enhanced resistance to one pathogen can correlate with elevated susceptibility to another, a phenomenon often attributed to shifts in the antagonistic SA–jasmonic acid balance discussed in Section “Salicylic Acid” below (Joumot-Catalino et al., 2006). More generally, the difference serves as a cautionary note about the potential pitfalls of predicting precise defense-related outcomes for different pathogens, even if dealing with the same host species.

In an intriguing twist, *Agrobacterium* has very recently been shown to exploit the WRKY network by co-opting several members of this transcription factor family to drive expression of a key gene on the T-DNA. The three major *Agrobacterium*-derived cancer-causing transgenes encode enzymes that direct the production of the phytohormones auxin and cytokinin (Lacroix and Citovsky, 2013a). Zhang et al. (2015) discovered that although the auxin-production genes *IaaH* and *IaaM* are constitutively expressed in *Arabidopsis*, the promoter for the cytokinin synthesis gene *Ipt* contains several W-boxes and is activated by the mutually interacting trio of WRKY18, WRKY40 and WRKY60. *WRKY40* and *WRKY60* are induced within 2 h of *Agrobacterium* infection, while *WRKY18* is turned on slightly later. WRKY40 binds directly to the *Ipt* promoter, and its ability to activate expression is synergistically enhanced by auxin. Predictably, mutants deficient in any of the three *WRKY* genes form smaller tumors than wild-type plants (Zhang et al., 2015). These three host factors apparently normally function to dampen host defenses to bacterial and fungal pathogens, perhaps as part of the plant’s feedback mechanism to prevent the deleterious effects of over-reaction to a pathogen (Pandey et al., 2010). The findings of Zhang et al. (2015) reveal that *Agrobacterium* has evolved to capitalize on this host self-defense strategy. Given the complex and varied repertoire of steps choreographed by the large WRKY superfamily, it seems likely that other family members may also be unwitting partners in the Agrobacterial thrust for control of its host’s metabolism.

## Contributions of Cytoskeletal Dynamics and Membrane Trafficking to the Regulation of Defense Responses

Genetic dissection of the molecular mechanisms underlying PTI and ETI has led to several illuminating discoveries linking plant immunity to intracellular trafficking and cytoskeletal dynamics. Both of these fundamental cell biology processes are critical and well-documented facets of mammalian innate immune responses and bacterial pathogenesis (reviewed in Day et al., 2011), but represent under-studied and exciting new areas of research with respect to plant defenses. In particular, recent findings specific to EFR are likely to be of potential interest to researchers interested in the host responses to *Agrobacterium*.

### Actin Dynamics

The efficiency of *Agrobacterium*-mediated transient transformation is reduced in actin-deficient roots and in cultured tobacco cells treated with actin microfilament or myosin light chain kinase inhibitors (Gelvin, 2012). Furthermore, exposure of *Arabidopsis* cotyledons to *Agrobacterium* causes an increase in actin filament density, without a concomitant change in the bundling of those filaments, within 6–9 h post-inoculation (Henty-Ridilla et al., 2013). These data are consistent with a growing appreciation for the involvement of actin dynamics in plant defenses. In light of recent discoveries in other host–pathogen interactions summarized here, the effects of *Agrobacterium*-induced changes in the host cytoskeleton on the progression of the infection is a question that warrants further investigation.

Changes in actin dynamics have been associated with both PTI and ETI in *Arabidopsis*. Susceptibility to *P. syringae* pv.* tomato* is increased upon pharmacological disruption of host actin filaments (Henty-Ridilla et al., 2013; Kang et al., 2014). Among the consequences that contribute to this outcome are defects in the actin-dependent protein transport and movements of endocytic vesicles required for immune system function (Kang et al., 2014). The *P. syringae* effector HopW1 promotes virulence by reducing the density of the actin filament network (Kang et al., 2014). Independently, Henty-Ridilla et al. (2013) also noted a change in actin bundling late in the infection only with a T3SS-competent pathogenic strain of this bacterium and that was hence attributed to effector-triggered events.

Using reverse genetics, Tian et al. (2009) identified the *Arabidopsis* actin depolymerizing factor 4 (ADF4) as essential for effector-specific HR. The sensitive phenotype of an *adf4* mutant could be partially rescued by exogenous application of an actin depolymerizing agent, supporting the claim that actin dynamics *per se* are required. In contrast with fungal and oomycete infections, in which ADF4 contributes to a block in pathogen entry, ADF4-dependent protection against the bacterial pathogen is linked to MAPK signaling (Tian et al., 2009; Porter et al., 2012). Taken together, the observation that ablation of *ADF4* specifically compromises resistance conferred by recognition of one specific bacterial effector, yet results in a reduction in expression of a PTI-specific target gene, provides additional evidence for coordinated regulation of PTI and ETI (Porter et al., 2012).

Subsequent experiments documented transient PTI-associated increases in actin filament density in *Arabidopsis* cotyledons as early as 3 h after challenge with bacterial or fungal pathogens, and with flg22 or chitin but not elf26; the flg22-induced changes required FLS2 as well as the co-receptors BAK1 and BIK1 (Henty-Ridilla et al., 2013). In contrast, in hypocotyls, high spatial and temporal resolution microscopic imaging of cortical actin filament architecture revealed EFR/BAK1/BIK1- and ADF4-dependent changes in single filament turnover within minutes following elf26 treatment (Henty-Ridilla et al., 2014). These changes lead to a rapid increase in the stability and hence overall number of actin filaments. Significantly, several hallmarks of elf26-triggered, but not chitin-triggered, PTI are dependent on ADF4 function; callose deposition and transcriptional changes downstream of CDPKs are disrupted in the *adf4* mutant, while MAPK-mediated responses to elf26 are unaffected. These findings strongly implicate ADF4 as an elf26-specific mediator of actin rearrangements and other downstream innate immune responses.

Among the other potential roles for actin dynamics in mediating host resistance is the regulation of stomate closure (Day et al., 2011). These openings serve as an important portal for bacterial entry into the host apoplast (Zeng et al., 2010). Both PAMPs (through their cognate PRRs) and effectors can trigger stomatal closure as an early line of host defense (Melotto et al., 2006; Zeng et al., 2010), and alterations in the arrays of actin filaments within the guard cells are associated with changes in stomate aperture (Gao et al., 2008; Day et al., 2011). To our knowledge, there is no published information on the stomatal response to *Agrobacterium*, although the elf18 peptide from *E. coli* can trigger closure in *Arabidopsis* (Zeng and He, 2010). As discussed below (see Pathogen Manipulation of Plant Hormone Responses), certain *P. syringae* strains have the ability to suppress closure (Melotto et al., 2006). Given that the guard cell transduction pathway mediating closure involves SA (Zeng and He, 2010), an early target of *Agrobacterium* intervention (see Modulation of Host Hormonal Responses by *Agrobacterium*), it seems plausible that *Agrobacterium* also has the ability to thwart the stomatal barrier.

### Membrane Trafficking

Membrane trafficking has also emerged as having important consequences for defense signaling. Within 1 h of ligand binding, FLS2, but not EFR, is internalized by endocytosis; *bak1-4* mutants bind flg22 with wild type-like efficiency but are inhibited in endocytosis of the bound ligands (Chinchilla et al., 2007). This cytoskeletal-dependent process results in localization of the PRR to late endosomal compartments called multi-vesicular bodies (Robatzek et al., 2006; Spallek et al., 2013). Ligand-bound endocytosed FLS2 is ultimately degraded, but this turnover is significantly slower than the internalization, leading to accumulated intracellular pools of activated receptor (Robatzek et al., 2006). In mammalian innate immunity, the analogous PAMP-responsive Toll-like receptors initiate certain signaling pathways from the endosome (McGettrick and O’Neill, 2010); whether the internal flg22-FLS2 pools are similarly functional is an open and potentially important question (Smith et al., 2014). Genetic ablation of the endosomal sorting complex required for transport (ESCRT-1) reduces FLS2 re-localization without affecting overall endocytic trafficking; the mutant plants are significantly more susceptible to colonization by *P. syringae* and are defective in flg22-induced stomatal closure, but not in other flg22-triggered responses such as oxidative burst, MAPK activation or callose deposition (Spallek et al., 2013). Pharmacological interference with the molecular machinery responsible for intracellular trafficking provided an additional approach to experimentally decouple various flg22-elicited responses. Collectively, the data to date suggest that ligand-triggered internalization and degradation of FLS2 desensitizes cells, potentially mitigating the costs associated with constitutive activation of FLS2-mediated defenses (Smith et al., 2014).

Genetic screens for EF-Tu-insensitive mutants and for FLS2-interacting partners uncovered several proteins involved in endoplasmic reticulum quality control and trafficking. Mutants deficient in the former process specifically abrogate EFR-mediated, but not FLS2-dependent, responses by abolishing accumulation of EFR (Li et al., 2009; Lu et al., 2009; Nekrasov et al., 2009; Saijo et al., 2009). At the same time, alterations in reticulon-like proteins that interact with FLS2 impair its accumulation at the plasma membrane and consequently FLS2-dependent signaling; FLS2 retention in the ER and its glycosylation, but not its stability, are affected (Lee et al., 2011). The dependence of EFR but not FLS2 on endoplasmic reticulum-associated folding factors for biogenesis is consistent with the notion that EFR may have evolved more recently than FLS2 and thus may not yet have undergone selection for high protein stability (Nekrasov et al., 2009; Saijo et al., 2009). It remains to be seen, however, whether this relative instability could also have mechanistic significance if, for example, different EFR conformers activate different, and separable, branches of the downstream signaling pathway (Saijo et al., 2009).

Unexpectedly differential fates for the two closely related receptors were also discovered through a yeast two-hybrid screen for BAK1 interactors. The results of this screen led to the finding that FLS2, but not EFR, is subject to polyubiquitination by PUB12 and PUB13, two E3 ubiquitin (Ub) ligases that are activated by BAK1-mediated phosphorylation. Upon flg22 binding to FLS2, the constitutively assembled PUB12/PUB13/BAK1 complex is recruited to FLS2. BAK1 then phosphorylates and thereby activates the Ub ligases, which rapidly ubiquitinate FLS2 but not BAK1 or BIK1. FLS2 ubiquitination appears to be unlinked to its internalization, but as expected modulates the ability of FLS2 to confer immunity; a *pub12/pub13* double mutant exhibits significantly higher resistance to *P. syringae* challenge (Lu et al., 2011).

These differences between FLS2- and EFR-centered intracellular events underscore again the need for caution when extrapolating from other, better-characterized plant pathogens to *Agrobacterium*. Despite the existence of multiple PAMPs and hence the possibility of functionally redundant or additive modes of elicitation by a given pathogen, at least some facets of the defenses induced in *Arabidopsis* by *P. syringae* appear to rely solely on FLS2 (Zeng and He, 2010). Conversely, as noted previously, *Agrobacterium* is detected primarily through EFR. In certain endoplasmic reticulum-quality control mutants in which EFR-mediated signaling is not completely abolished, the downstream outputs (MAPK signaling, callose deposition, and ROS production) are uncoupled, i.e., only partially and differentially impaired. Significantly, the characterization of these mutant plant lines has led to the proposal that there may be differences between FLS2 and EFR in the order of these post-recognition defense events (Lu et al., 2009).

## Hormone Regulation of Systemic and Local Plant Immunity

Plant hormones typically act as mediators between external input and internal responses on both a systemic and intracellular level, often by influencing developmental processes. Their wide-ranging effects require tight control, as evidenced by the extensive antagonistic and synergistic cross-regulation that occurs among them. Three plant hormones, ethylene, JA, and SA are known as classic defense regulators, though other abiotic and developmentally induced hormones, including auxin, cytokinins, abscisic acid and giberellins, are also thought to be involved (Robert-Seilaniantz et al., 2011). In the sections below, we summarize briefly the roles of each of the three major defense hormones, before expanding on the pathways downstream of SA that, based on existing data, are most likely to represent potential foci of subversion by *Agrobacterium*.

### Ethylene

Ethylene is a gaseous olefin that is critical for plant developmental processes including fruit ripening, senescence, and leaf abscission (Schaller, 2012). It accumulates in response to herbivore damage and mechanical wounding and is believed to fine-tune the balance between JA and SA-induced defenses (described below), as it can alternately reinforce or repress either in a context-dependent manner. *ein2* and *ein1* mutants, which overproduce ethylene but are characterized by ethylene insensitivity, exhibit increased disease susceptibility compared to wild-type plants (Guzmán and Ecker, 1990; Boutrot et al., 2010; Mersmann et al., 2010). Several lines of evidence indicate that ethylene contributes significantly to PTI. First, expression of the *FLS2* receptor gene is controlled by the binding of an ethylene response transcription factor, EIN3, to its promoter. Conversely, flg22 treatment induces both *EIN3* expression and ethylene synthesis (Felix et al., 1999; Chen et al., 2009; Boutrot et al., 2010). Thus, ethylene may play a role in regulating the positive feedback of *FLS2* accumulation observed in response to EF-Tu and flg22 stimulation (Zipfel et al., 2006; Robert-Seilaniantz et al., 2011). Significantly, although *ein2* mutants are less sensitive to elf18 as well as flg22, the mechanisms underlying these dependencies are different; in contrast to *FLS2*, both *EFR* expression and accumulation of the encoded receptor are unaffected by the *EIN2* deficiency (Tintor et al., 2013). Ethylene signaling is also required for the FLS2-triggered oxidative burst (Mersmann et al., 2010). Furthermore, MPK6, activated by FLS2-triggered PTI, has a role in stabilizing ACS2 and ACS6, enzymes necessary for ethylene biosynthesis (Liu and Zhang, 2004). These results suggest a close connection between the initial events that trigger PTI and ethylene-induced plant immunity (Liu et al., 2013).

### Jasmonate

Jasmonate is a lipid-derived signaling molecule ubiquitous to plants, animals, fungi, and some algae (Thaler et al., 2012). In plants, JA has roles in root growth inhibition, tuber formation, touch-mediated responses, flower development, and senescence (Wasternack, 2007). JA synthesis occurs via a linoleic acid precursor in response to herbivores and mechanical wounding, and both JA- and ethylene-dependent responses are required for resistance to necrotrophic pathogens. Some genes regulated by jasmonic acid are also regulated by ethylene, so many discussions of hormonal defense pathways tend to invoke JA and ET pathways together. For example, at least one transcription factor, ERF1*,* requires both hormones to activate defense gene transcription (Glazebrook, 2005). Moreover, EIN2, a necessary component of the ET-induced pathway can itself restore both ethylene and jasmonic acid signal responses in ethylene insensitive plants (Alonso et al., 1999).

### Salicylic acid

The phenolic acid SA can be synthesized through two major pathways in plants, including one that uses phenylalanine as a precursor (Seyfferth and Tsuda, 2014). However, defense response-induced SA is typically only synthesized through the isochorismate (ICS) pathway, and a key enzyme (ICS1), encoded by the gene *SID2*, is often a target for SA regulation by downstream transcription factors (Wildermuth et al., 2001; Wang et al., 2006). The conservation of ICS1 in algae and bacteria suggests that the pathway may have originated from a plastid through an evolutionary endosymbiotic event (Wildermuth et al., 2001). Such a relationship could explain one way that certain pathogens, e.g., *P. syringae*, have evolved mechanisms to subvert plant hormone-mediated defenses. A well-known example is the bacterial NahG hydrolase that metabolizes SA. SA is critical for the induction of systemic acquired resistance, or SAR (Vlot et al., 2009). As initially described more than half a century ago, SAR results in enhanced resistance to secondary infection in locations far from the primary site of pathogen exposure (Ross, 1961). *nahG*-expressing plants have long been used in plant immunity research because they are unable to accumulate SA, induce SAR or express the pathogenesis-related (*PR*) genes that act as hallmarks and mediators of SAR. It should be noted, however, that pleiotropic effects caused by *nahG* overexpression have been reported (Heck et al., 2003). Similarly, *sid2-2* mutant plants with a defective ICS1 enzyme are unable to induce PR1 (Wildermuth et al., 2001) and conversely, exogenous application of SA or its functional analogs is sufficient to trigger PR gene expression (White, 1979).

The bioactive form of SA can only act on a local level or through phloem transport. Long-distance SAR induction is mediated by the volatile and biologically inactive form, methyl SA; interestingly, the extent to which SAR requires MeSA is light-dependent (discussed in more detail below). Perhaps the most intriguing recent discovery in this field is the existence of “transgenerational” SAR, in which enhanced resistance was observed in the progeny of primed hosts, even after a biotic stress-free generation. This epigenetic phenomenon is due to changes in histone modification and DNA methylation state, rather than hormone levels (Luna et al., 2012). Future exploration of this imprinting might prove fruitful in the development of disease-resistant seed stock.

The ethylene, SA and JA hormonal pathways work with other defense responses to orchestrate different defenses against biotrophic and necrotrophic pathogens (Glazebrook, 2005). Since biotrophic pathogens acquire sustenance from live plant tissue and nectrophic pathogens feed on dead plant tissue, the HR responses that result in plant cell apoptosis would provide resistance against biotrophic pathogens and encourage necrotrophic pathogen growth. Generally speaking, the SA-dependent pathway is elicited in defenses against biotrophic pathogens such as *Agrobacterium*, while as mentioned above, the ET/JA-dependent pathways are activated in the presence of necrotrophic pathogens (Glazebrook, 2005). However, more detailed multi-mutant analysis has revealed that all three hormonal pathways positively regulate defenses induced by both necrotrophic and biotrophic pathogens to different degrees (Tsuda et al., 2009). These data suggest a model in which an unspecialized and highly interconnected network may enable plants to maximize survival upon simultaneous attack by multiple pathogens on a single plant (Bar-Yam et al., 2009).

Evolutionary evidence suggests that JA–SA antagonism dates to the last common ancestor of land plants, although this antagonism may have arisen multiple times. In animals, an SA-derivative (acetyl salicylic acid, or aspirin) also inhibits JA-like prostaglandins, blocking platelet aggregation and pain transmission (Thaler et al., 2012). In plants, low concentrations of JA and SA can synergistically increase levels of *PR1* gene expression, but high levels work antagonistically, inducing ROS production and cell death. The regulatory mechanisms mediating this antagonism are complex, involving proteins (e.g., MAPK4) that suppress SA signaling but are required for the JA-induced pathway, and others (PAD4 and EDS1) that act to repress the JA/ET pathways while simultaneously increasing the SA-induced defense pathway (Loake and Grant, 2007). Like JA, the ethylene signal transcription factor EIN3 can also decrease SA levels by directly binding to the *SID2* promoter sequence and down-regulating gene expression (Chen et al., 2009).

#### NPR1 Mediates SA-Responsive Modulation of Defenses

NPR1 is a critical mediator of SA action and nexus of SA/JA crosstalk. This positive regulator of SA-induced defenses is responsible for SA-responsive transcriptional reprogramming on a global genomic level through both direct and indirect mechanisms. At the same time, NPR1 acts through a negative feedback loop to limit SA accumulation (Shah, 2003). The *NPR1* (non-expresser of PR genes 1) gene was first identified through a screen of mutants deficient in SAR-induced PR gene expression (Cao et al., 1994). Further genetic screening for SA-insensitive mutants repeatedly revealed only mutants of *npr1* alleles, suggesting that either SA directly regulates NPR1 or that there are functionally redundant signaling factors between SA recognition and NPR1 activation (Dong, 2004). NPR1 expression occurs at a low constitutive level and does not change dramatically upon SA induction or pathogen infection. Its overexpression confers a protective effect but does not correlate with constitutive expression of *PR* genes (Ryals et al., 1997; Cao et al., 1998; Durrant and Dong, 2004), suggesting that NPR1 is primarily regulated post-transcriptionally.

Cytosolic NPR1 is held inactive as oligomers and released NPR1 monomers translocate to the nucleus, where they associate with the TGA family of transcription factors; this complex binds to SA-responsive *cis*-acting promoter elements to induce PR gene expression (Dong, 2004). How SA activates NPR1 remains an intriguing, if challenging, open question. Much of what we know thus far comes from the Dong and Després labs, whose mechanistic studies have slowly unraveled the mechanisms of NPR1 activation over the past decade. One recent study demonstrated that two NPR-family proteins, NPR3 and NPR4, bind SA with differing affinities and act as adaptors for proteasomal degradation of NPR1. In a yeast two-hybrid system, exogenous SA promoted NPR1-NPR3 association while disrupting the interactions between NPR1 and NPR4 (Fu et al., 2012). These and other data led these authors to propose a model in which basal levels of NPR1 are modulated upon ETI by the gradient of SA that develops in and around the site of infection. In this model, high SA concentrations in the center of the HR lesion facilitate NPR3-mediated degradation of NPR1, allowing programmed cell death to proceed, while lower SA levels at the margins enable NPR1 accumulation which inhibits the spread of the HR and promotes SAR. In the absence of pathogen challenge, the high-affinity binding of NPR4 with SA relieves the constitutive NPR4-directed turnover of NPR1 that prevents inappropriate defense activation. Other data and models, however, argue that NPR1 binds SA directly, leading either to a conformational change that induces oligomer disassembly and thereby releases auto-inhibition of NPR1 (Wu et al., 2012) or to diminished inhibitory interactions between NPR1 and the negative defense regulator NIMIN2 (Maier et al., 2011). Additionally, sequential interactions with various members of the NIMIN protein family may enable NPR1 to respond to differential concentrations of SA, preventing inadvertent PR activation (Hermann et al., 2013).

While SA-dependent activation of NPR1 seems to be a critical node in SAR regulation, *NPR1* expression may itself be modulated by other defense genes. Its promoter contains W-boxes for WRKY binding (Yu et al., 2001), and conversely, NPR1 directly targets and upregulates expression of many *WRKY* genes. Elucidation of NPR1 targets has proven difficult because NPR1 requires SA activation, and simple transcriptome analysis of SA-induced cells would yield many indirect or non-NPR1 targets. Using a transgenic plant line expressing *NPR1* under glucocortecoid-inducible promoter control and comparing SA-induced changes in the transcriptomes of a protein synthesis-inhibited and a non-inhibited sample, Wang et al. (2006) identified candidate genes that were directly regulated by NPR1. Of these, mutant analysis revealed both positive (e.g., WRKY18) and negative (e.g., WRKY58) regulators of SAR. NPR1 also targets WRKY70 and its functional homolog, WRKY54, which both act as positive regulators of SA-mediated gene expression and resistance, but repress SA biosynthesis through ICS1, thus regulating SA/JA cross-talk.

### Pathogen Manipulation of Plant Hormone Responses

Pathogens may modulate host hormone responses to increase the likelihood of successful infection or, as in the case of biotrophic pathogens, to create a more favorable environment for long-term survival. One of the most direct examples of modulation comes from the hemibiotrophs *P. syringae* pv. *tomato* (*Pst* DC3000) and pv. *maculicola* ES4326, which repress SA accumulation in *Arabidopsis* through a JA analog and phytotoxin, coronatine (Bereswill et al., 1994; Kloek et al., 2001; Brooks et al., 2005). Mechanistically, coronatine upregulates NAC transcription factors. In guard cells, this prevents the stomatal closure defense response (Melotto et al., 2006), while in neighboring leaf cells, it suppresses *ICS1* and induces the SA-metabolizing genes *SAGT1* and *BSMT1* (Zheng et al., 2012). Thus, certain strains of *Pseudomonas* take advantage of the plant’s natural hormone JA, and its antagonistic relationship to SA, by producing an effector that mimicks JA, thereby suppressing SA-mediated host defenses necessary for establishing local and systemic bacterial resistance (Brooks et al., 2005).

#### Modulation of Host Hormonal Responses by *Agrobacterium*

Several lines of evidence indicate that *Agrobacterium* also engages in hormonal dueling to attenuate host defense responses, although this story is far from fully resolved. Extensive transcriptome profiling of JA/SA/ethylene and auxin-induced genes in* Agrobacterium*-inoculated *Arabidopsis* stems over three timepoints (3 hpi, 6 dpi, 35 dpi) revealed that a number of auxin and ethylene-signaling genes were upregulated in response to the virulent *Agrobacterium* C58 strain, while only a few auxin-related genes responded to inoculation with the avirulent T-DNA deficient *Agrobacterium* strain GV3101 (Lee et al., 2009). Since ethylene has been demonstrated to suppress *Agrobacterium* virulence, the increase in host ethylene levels upon infection may contribute to the plant’s ability to combat agrobacterial infection at a relatively early stage (Nonaka et al., 2008; Lee et al., 2009). Indeed, although *Agrobacterium* lacks the ACC deaminase used by other plant-associated bacteria to enzymatically cleave the ethylene precursor, engineering the bacteria to express this enzyme enhances transformation efficiency (Nonaka and Ezura, 2014).

In contrast, the relationship between *Agrobacterium* infection and SA-mediated defenses defies simple characterization based on known paradigms for SA activity. Tomato seedlings respond to *Agrobacterium* exposure by altering which pathway is utilized for SA synthesis (Chadha and Brown, 1974). On tobacco leaves, 48 h of exposure to *Agrobacterium* is sufficient to dampen the SA production elicited by subsequent *P. syringae* inoculation, and confers resistance both to *P. syringae* colonization (Rico et al., 2010) and to tobacco mosaic virus by a mechanism that is at least partially SA-dependent (Pruss et al., 2008). Interestingly, neither of these effects requires Ti-plasmid encoded functions. In *Arabidopsis* stems, SA accumulates in C58-inoculated plants at 6 days, but not at 3 h, post-infection, suggesting a role for SA in regulating late defenses (Lee et al., 2009). In contrast, an earlier study had found that within an hour after bacterial attachment, SA accumulation is reduced by 40% and *PR* genes are down-regulated in *Arabidopsis* roots infected with the avirulent strain GV3101 (Gaspar et al., 2004). The *Agrobacterium*-triggered activation of MPK4 as rapidly as 5 min post-inoculation (Djamei et al., 2007) may be one of the ways this plant response is attenuated, as MPK4 has been shown to negatively regulate both pathogen-induced SA accumulation and ROS production (but not callose deposition; Berriri et al., 2012). Since SA directly inhibits expression of the *Agrobacterium* virulence genes required for T-DNA delivery (Yuan et al., 2007; Anand et al., 2008), down-regulation of SA accumulation in the first hour(s) of infection would enable the bacterium to initiate the transformation process. Indeed, tumor growth is significantly higher in mutant plants with low endogenous SA levels (*nahG*, *eds1*, *pad4*), and lower in mutant plants with high endogenous SA levels (*npr1*, *cpr5*; Yuan et al., 2007; Anand et al., 2008; Lee et al., 2009). Paradoxically, stems from *sid2* mutants have been reported to exhibit wild-type amounts of tumor formation (Lee et al., 2009). Furthermore, neither *ICS1* expression nor *PR1* gene expression (typically induced by elevated SA levels) is affected by either virulent or avirulent agrobacterial strains at the time points examined, although genes implicated in SA methylation and other *PR* genes are upregulated after T-DNA integration (Lee et al., 2009).

Taken together, these data implicate ethylene and an NPR1-independent function for SA in protecting the host against* Agrobacterium*, likely in part by attenuating the bacterial virulence machinery at early stages of the infection process (Yuan et al., 2007; Anand et al., 2008; Lee et al., 2009). Conversely, by manipulating hormonally regulated plant defense pathways, *Agrobacterium* is able to confer resistance to subsequent pathogen challenge.

## Host Physiology Influences Defense and Hormonal Pathways

### Circadian Effects

Given that both hormones and light control many aspects of plant life, it is not surprising that the defense and hormonal pathways interact and crosstalk with the plant circadian clock in a variety of ways (Robertson et al., 2009). In *Arabidopsis*, *P. syringae* infections during morning and midday elicit higher SA levels and greater defense responses than in the evening (Griebel and Zeier, 2008). The circadian-regulated PHT4;1 (phosphate transporter 4;1), also named ANTR1, acts upstream of the SA-induced pathway (Wang et al., 2011). JA accumulates in a circadian-regulated fashion that coincides with the feeding patterns of *Trichoplusia ni* insects, allowing *Arabidopsis* an advantage over herbivores by perpetually mounting a timely resistance against attack (Goodspeed et al., 2012). Ethylene production peaks at midday in *Arabidopsis;* the* ACS* genes necessary for ethylene biosynthesis are both circadian- and light-regulated, although mutants with aberrant ethylene production exhibit normal circadian control over cycles of growth (Thain et al., 2004). Finally, circadian gating of light-responsive, hormone-triggered stomata opening (Robertson et al., 2009) may have important implications for pathogen entry.

The circadian clock also affects hormone-independent defense pathways, although this is less well-studied. Circadian clock component transcription factors CCA1 (circadian clock associated 1) and LHY (late elongated hypocotyls) directly regulate defense activation independent of SA-induced defense responses (Schaffer et al., 1998; Wang and Tobin, 1998; Zhang et al., 2013a). A number of defense-related genes including *PHT4;1* and the *PCC1* (pathogen and circadian controlled) gene, which plays a role in defenses specifically against virulent oomycetes, are under CCA1 transcriptional control (Sauerbrunn and Schlaich, 2004; Wang et al., 2014). CCA1 and LHY synergistically affect both PTI and ETI, in part by regulating stomatal control. Significantly, *P. syringae* infection or the PAMP flg22 feed back to affect the circadian clock, thus demonstrating cross-talk between innate defenses and the circadian clock (Zhang et al., 2013a).

Involvement of the circadian clock in defenses enhances the plant’s ability to anticipate potential infection and to mount defenses accordingly (Eriksson and Millar, 2003). In *Arabidopsis*, the circadian clock modulates PTI-related responses to anticipate dawn infection of *P. syringae* pv. *tomato* DC3000 by up-regulating levels of downstream responders, including the MKK4/5-MAPK3/6-WRKY22 protein module and WRKY29 (Bhardwaj et al., 2011). Similarly, in the case of the fungal pathogen *Hyaloperonospora arabidopsidis*, circadian-regulated defense gene activation increases at dawn and decreases at dusk, consistent with the fact that *H. arabidopsidis* releases spores at dawn (Wang et al., 2011). As these examples illustrate, it is clear that plants with functional circadian rhythms may have an advantage in combating pathogenic attack by anticipating pathogen infection and priming defenses at peak-infection times to maximize growth time and resources (McClung, 2011). There are as yet no published reports on the impact of day length or time-of-day of inoculation on the outcome of *Agrobacterium* infection. The data reviewed here suggest, however, that such effects would not be surprising, and would point to the importance of early defenses in determining the success of the agrobacterial assault.

### Developmental Stage of the Plant

More mature hosts are often more resistant than younger plants, a phenomenon that has been referred to generically as age-related resistance (ARR). A variety of forms of resistance are encompassed by this term; they vary with host species and with pathogen, and can be either broad-spectrum or specific to one pathogen, pathovar, or strain. Lasting resistance emerges at multiple major life cycle transitions, such as juvenile to adult, flowering or upon the onset of senescence, although neither flowering nor senescence *per se* appears to be required or sufficient for ARR (Carella et al., 2015). In some cases, race-specific ARR reflects the induction of R genes at a particular point in the plant’s development (Develey-Riviere and Galiana, 2007). Hormonal pathways, central as they are to various developmental processes, also contribute to ARR. For example, although ARR is distinct from SAR, SA accumulation is required (Kus et al., 2002), and both developmental and pathogen modulation of SA may be key determinants of ARR (Carella et al., 2015). The developmental transition from susceptibility to resistance includes the accumulation of anti-microbial compounds, possibly SA itself, in the intercellular fluids (reviewed in Carella et al., 2015), although the mechanisms of resistance differ between *Arabidopsis* and tobacco (Kus et al., 2002; Develey-Riviere and Galiana, 2007). Given the apparent centrality of SA in the *Agrobacterium*-host interchange, these observations raise the possibility that the outcome of *Agrobacterium* infection may be exquisitely sensitive to ARR, and that different labs could obtain distinctly non-concordant results depending on the precise developmental stage of the host at the time of infection.

## Environmental Influences on Plant Defenses and Microbial Virulence

### Light-Dependent Effects

Though the circadian clock is entrained by the presence and absence of light to regulate plant defenses, light stimulus can itself affect plant responses to pathogen encounters (Roden and Ingle, 2009). The strength of both *PR* gene induction and the HR response depend on the presence of light (Genoud et al., 2002; Chandra-Shekara et al., 2006). Moreover, the time-of-day dependence of defenses is at least partially due to the availability of light and phytochrome photoperception during local and systemic defense induction, independent of any circadian-regulated stomatal control (Griebel and Zeier, 2008). For example, both accumulation and activation of the radish-derived defense regulator Raphanusanin require light (Moehninsi et al., 2014). More generally, Sano et al. (2014) recently showed that 30% of the genes up-regulated upon flg22 perception require light, and specifically photoelectron flow, for that induction; several of those genes are involved in SA biosynthesis, and flg22-treated plants accumulated more SA in the light than in the dark. In this study plants were illuminated for 4 h prior before the flg22 elicitation. Overall, there was significant overlap between the pool of flg22-responsive genes and those that were light-dependent or light-repressed.

Thus, the success of a pathogenic infection may be partially determined by the prior (or subsequent) light exposure, and hence time of day of the inoculation, which can impact laboratory research experiments on plant defenses (Roden and Ingle, 2009). Perhaps the clearest illustration of this principle to date is the influence of light on the requirements for SAR signaling. Several lines of evidence in multiple host species have implicated methyl salicylate (MeSA), a volatile form of SA, as the signal molecule that is translocated through the phloem to distal leaves, where it is converted to active SA (Park et al., 2007). However, Attaran et al. (2009) showed that *A. thaliana* mutant lines deficient in methyl SA production are nonetheless able to mount SAR in leaves distal to the site of pathogen inoculation. Liu et al. (2011) resolved this apparent conflict by discovering that MeSA is essential for SAR development in plants infected late in the day but not in the morning; the key determinant was shown to be the length of time of light exposure subsequent to the inoculation.

While light stress induces excess excitation energy (EEE) relative to that needed for normal physiological photosynthetic activity, pathogen infection can also induce EEE by affecting photosynthetic rates. Moreover, EEE-induced plant responses share similarities to defense responses including increases in ROS, SA-induced signaling through EDS1 and PAD4, and programmed cell death. Interestingly, plants acclimated to high intensities of light also display high levels of resistance against pathogens, similar to the effect of SAR in resistance to secondary infections (Roden and Ingle, 2009).

Light also seems to have an effect on the virulence of some pathogens. In *Agrobacterium*, the presence of constant light during co-cultivation with host plants correlates with higher levels of T-DNA transfer, potentially by affecting attachment to plant cells and/or plant cell’s ability to take up the *Agrobacterium* T-DNA (Zambre et al., 2003). Additionally, in the *Dendrobium* orchid and *Agrobacterium* model, synthesis of the virulence gene inducer coniferyl alcohol is stabilized in the presence of light (Nan et al., 1997). Paradoxically, the presence of light has been reported to decrease *Agrobacterium* accumulation of flagellar proteins FlaA and FlaB, reducing bacterial motility, attachment to plant cells, and bacterial virulence on cucumber (Oberpichler et al., 2008).

### Temperature-Dependent Effects

In addition to light, SA-mediated responses and susceptibility to pathogen attack are affected by temperature. Cheng et al. (2013) recently demonstrated that PTI, as measured by expression of the MAPK target genes *WRKY29* and *FRK1* in response to flg22, is most activated in *Arabidopsis* at temperatures between 23 and 32^∘^C (with an optimum of 28^∘^C). In contrast, activation of ETI and resultant cell death by inducible expression of a bacterial effector transgene peaked at 16^∘^C and was significantly less pronounced at 28^∘^C or higher. In tobacco, the failure of the HR or SAR at elevated temperatures is correlated with a lack of *PR* gene induction, which can be overcome by exogenous application of SA (Yalpani et al., 1991). The preferential activation of ETI at low ambient temperatures correlates well with the temperature range at which bacterial secretion systems and effector production are optimally functional (Cheng et al., 2013). In the case of *Agrobacterium*, we and others have shown that elevated temperature (28^∘^C) prevents biogenesis of the VirB/VirD4 Type IV secretion system responsible for export of the T-DNA and several virulence proteins (Banta et al., 1998; Baron et al., 2001). Conversely, bacterial proliferation, and hence synthesis of PAMP elicitors, thrive at the elevated temperature range at which the PTI is most responsive (Cheng et al., 2013).

## Areas Ripe for Future Research

In conclusion, the success of pathogenic infection on plants results from a complex network of interwoven interactions among host recognition/response systems, plant hormonal pathways, circadian rhythms, light perception, and other plant-specific events such as host developmental stage, as well as bacterial and fungal virulence factors. For practical as well as historical reasons, much of the research to date on plant defenses has focused on a rather limited subset of pathogens and host species. The great majority of these investigations have utilized leaf tissue and/or seedlings as a model system. Yet as the plant tissue exposed to the remarkable microbial richness of the rhizosphere, roots represent perhaps the most relevant site for studying many *in situ* host–microbe interactions (De Coninck et al., 2014). Recent work in the Ausubel lab has confirmed that *Arabidopsis* roots mount complex, highly choreographed, tissue-specific responses to PAMP/MAMP elicitation (Millet et al., 2010). Some of these responses are ethylene-dependent, and some are suppressed by coronatine mimicking of JA but, unlike in leaves, in a manner that is independent of SA–JA antagonism. Significantly, these authors detected no root response to the EF-TU-derived elicitor elf26 (Millet et al., 2010), implying that this host lacks a critical mode of surveillance for *Agrobacterium* in the pathogen’s primary habitat.

Given that defense gene expression patterns are not always conserved in timing or magnitude among different host tissues, future efforts will be needed to explore the similarities and differences between the canonical model systems and the other “native” settings in which chance or deliberate host–microbe encounters occur. In nature, of course, every such encounter is perturbed by countless bystander microbes, some hoping to “cut in” to the dance while others are merely milling around, crowding the dance floor. While some bystanders may suppress host defenses, others induce defenses, priming the host for enhanced resistance to subsequent pathogen exposure. Indeed, this resistance forms the basis for one common assay for the capacity of a pure elicitor to incite basal defenses. The nature of the activated defenses will almost certainly vary depending on the partners tested, but many such studies to date have relied upon a single bacterial model, *P. syringae*. Deepening the pool of well-characterized host defense-pathogen relationships to include *Agrobacterium* will likely uncover previously uncharacterized PAMPs and their cognate receptors, as well as novel mechanisms by which the microbes suppress or thwart their hosts’ responses.

## Conflict of Interest Statement

The authors declare that the research was conducted in the absence of any commercial or financial relationships that could be construed as a potential conflict of interest.
